# Mediating Roles of Gratitude and Social Support in the Relation Between Survivor Guilt and Posttraumatic Stress Disorder, Posttraumatic Growth Among Adolescents After the Ya’an Earthquake

**DOI:** 10.3389/fpsyg.2018.02131

**Published:** 2018-11-05

**Authors:** Wenchao Wang, Xinchun Wu, Yuxin Tian

**Affiliations:** Beijing Key Laboratory of Applied Experimental Psychology, Faculty of Psychology, Beijing Normal University, Beijing, China

**Keywords:** survivor guilt, gratitude, social support, posttraumatic stress disorder, posttraumatic growth

## Abstract

**Objective:** This study aims to examine the mediating roles of gratitude and social support in the relationship between survivor guilt and posttraumatic stress disorder (PTSD) as well as the relationship between survivor guilt and posttraumatic growth (PTG).

**Methods:** The current study used self-report questionnaires to investigate 706 adolescent survivors of Lushan county three and a half years after the Ya’an earthquake. The structural equation model was used to evaluate the relations between survivor guilt, gratitude and social support in PTSD and PTG.

**Results:** The results indicated that survivor guilt had a positive effect on both PTSD and PTG. Gratitude partly mediated the relation between survivor guilt and both PTSD and PTG; social support partly mediated the relation between survivor guilt and PTG but not PTSD as well as the relation between gratitude and PTG.

**Conclusion:** Survivor guilt has a double-edged sword effect. Survivor guilt affects PTSD and PTG through gratitude, and it could affect PTG but not PTSD through social support. Gratitude decreases PTSD and increases PTG, whereas social support only increases PTG.

## Introduction

Earthquakes can usually increase the risk of psychological distress, such as posttraumatic stress disorder (PTSD; Wang et al., 2012; Fan et al., 2015), among adolescent survivors. However, trauma survivors also report positive changes in various aspects of their lives, such as changes in self-perception, interpersonal relationships and philosophy of life, which Tedeschi and Calhoun (1996) have collectively defined as posttraumatic growth (PTG).

As two typical posttraumatic reactions, PTG and PTSD can coexist in survivors following trauma exposure (Dekel et al., 2011). However, the relation between PTSD and PTG remains mixed in previous recent studies. Several studies argue a positive relation between PTSD and PTG (e.g., Wu et al., 2016), whereas other studies suggest a negative relation exists between these two posttraumatic reactions (e.g., Johnson et al., 2007). In addition, other researchers found the correlation between PTSD and PTG was not significant (e.g., Zhou et al., 2015). Hence, it is necessary to examine the predictive factors of PTSD and PTG (Dekel et al., 2011). The present study simultaneously examines the predictors of PTSD and PTG among adolescent survivors of the Ya’an earthquake.

Trauma exposure is undoubtedly a precondition for posttraumatic reactions, however, many trauma survivors have not reported PTSD (Goldenberg and Matheson, 2005) or PTG (Jin et al., 2014). Thus, other factors may affect posttraumatic stress, of which guilt serves as one crucial predictor (Pugh et al., 2015; Dekel et al., 2016). Guilt is a self-conscious affect and a moral emotion characterized by negative self-evaluation (Tangney and Dearing, 2002; Tangney et al., 2007). Individuals may experience guilt when they believe they have done something wrong or when they feel their actions have hurt others (Lee et al., 2001). According to Kohlberg’s theory of moral development (Kohlberg, 1971), adolescents typically have a conventional level of moral reasoning. At this level, an individual obeys rules and follows society’s norms. The adherence to rules and conventions is somewhat rigid, and a rule’s appropriateness or fairness is seldom questioned. Adolescents at the conventional level are more likely to blame themselves and feel guilty for illegal or immoral things they did in a traumatic event. Furthermore, Hoffman (2000) suggests that with the development of empathy, adolescents are more likely to feel virtual guilt. Adolescents will feel guilty, not only for they did something wrong, but also when they think they can do more to help others. In other words, adolescents are more likely to feel guilty about their own inaction during traumatic events. Therefore, it is necessary to study the effects of guilt on posttraumatic psychological response of adolescent.

Guilt is commonly found in war veterans, victims of violent crime and the perpetrators of traffic accidents (Lowinger and Solomon, 2004; Marx et al., 2010; Semb et al., 2011; Solomon et al., 2015). Niederland (1968) referred to the guilt caused by trauma as survivor guilt, and Lifton (1980) described an individual suffering from survivor guilt as one who has encountered, been exposed to, or witnessed death and has remained alive. However, some studies have found survivor guilt also exists in survivors of natural disasters (Grant et al., 1997; Krug et al., 1998), such as earthquakes (Carmassi et al., 2017). After the earthquake, many survivors struggle to make sense of fatal traumas and may experience survivor guilt. A person experiencing survivor guilt may have emotional distress and negative self-appraisal related to surviving the disaster while others did not. Survivor guilt occurs when survivors feel responsible for the death or injury of others, even if the survivor had no real power of influence in the situation (Tangney and Dearing, 2002).

In recent years, more psychologists have paid attention to the effect guilt has on PTSD and PTG (Lee et al., 2001; Pugh et al., 2015; Dekel et al., 2016). According to Birrer and Michael (2011), guilt is conceptualized as a multi-dimensional construct entailing negative affect and related cognitions. On the one hand, guilt is not conducive to the integration of individual post-traumatic core beliefs (Kubany and Manke, 1995). Survivors from the trauma may develop guilt behavior patterns, such as alexithymia, irritability and self-deprecation, which can lead to PTSD (Hutson et al., 2015). Some empirical studies have found that guilt is one of the predictive factors leading to the development of PTSD (Kubany et al., 1996; Beck et al., 2011; Norman et al., 2014). On the other hand, guilt is not always associated with negative mental reactions and may be adaptive in certain situations (Tangney and Dearing, 2002). Guilt is a typical moral emotion that has psychological adaptation functions (Tangney and Tracy, 2012). Guilt can increase one’s empathy, promote the establishment of good interpersonal relationships, and help individuals effectively cope with setbacks (Tangney et al., 2007). Thus, guilt may foster PTG in a person’s self-perception, interpersonal relationships and life philosophy. A recent study found that PTG could be facilitated by survivor guilt (Dekel et al., 2016).

Some studies had investigated the mediating factors in the effect of guilt on posttraumatic psychological response. For example, Lee et al. (2001) suggest that when survivors feel guilty, they may constantly reflect on what they have done in the traumatic event and reexamine or repetitively think about their beliefs before and after the trauma, so rumination may play an important mediating role in the relationship between guilt and PTSD/PTG. Kubany and Manke (1995) suggest that avoidant coping may form a pathway through which guilt could affect PTSD, possibly by preventing emotional processing and therefore the successful integration of a traumatic event with prior beliefs and experiences. Nevertheless, the predictive mechanisms for survivor guilt in relation to PTSD and PTG remain unclear. The broaden-and-build theory of positive emotions (Fredrickson, 2001) and the main-effect hypothesis of social support (Cohen and Wills, 1985) suggest that gratitude and social support may play a mediating role in the relation between survivor guilt and PTSD/PTG.

Also a moral emotion, gratitude is a positive emotion that gives the feeling of an appreciation for others (McCullough et al., 2004). Gratitude is promoted when individuals have more resources than others (McCullough et al., 2001), and a typical perception of those with survivor guilt is individuals considering themselves luckier than others or have more access to relief or survival opportunities during or after traumatic events (Matsakis, 1999). In other words, individuals with higher levels of survivor guilt may considering themselves more fortunate than others in the earthquake. This in turn makes them grateful for their fate and the help from others. Hence, survivor guilt may promote gratitude after stressful experiences.

In addition, the broaden-and-build theory of positive emotions (Fredrickson, 2001) emphasizes that gratitude is one of human’s evolved adaptation mechanisms that can in the moment broaden individuals’ “thought-action” repertoire and build some enduring personal resources over time (Fredrickson, 2001), gratitude may also have long-term survival benefits by making people more open-minded and flexible, and ultimately they can see and take advantage of opportunities in the environment better (Johnson and Fredrickson, 2005), which then enhances one’s adaptive activities and optimal experiences. Such processes can help trauma survivors form positive associations with traumatic cues and reestablish adaptive worldviews after trauma (Zhou and Wu, 2016). Thus, gratitude represents a common ingredient of PTG (Ruini and Vescovelli, 2013) that can lead to the realization of PTG (Peterson et al., 2008; Wu et al., 2014). Furthermore, gratitude can help survivors bounce back from negative emotional experiences and eliminate the physiological effects of negative emotions (Tugade and Fredrickson, 2004), implying gratitude is negatively associated with PTSD (Zheng et al., 2011) and positively associated with PTG (Zhou et al., 2014; Zhou and Wu, 2015).

Social support may be another important mediator of the relation between survivor guilt and PTSD/PTG (Eray et al., 2017; Jia et al., 2017). Analysis of the literature revealed that guilt is a moral emotion that can improve interpersonal relationships by contributing toward the happiness of others (Carnì et al., 2013). Thus, when individuals feel guilty, they present more altruistic behavior and receive higher levels of social support. Additionally, McCullough et al. (2002) suggest that grateful people tend to possess a worldview in which everything they possess is seen as a gift. When this positive worldview is applied to a relational context, gratitude can be regarded as a moral barometer that is sensitive to changes in social relationships, particularly the benefits received from another moral agent. Thus, it is likely that gratitude can be predictive of social support during PTSD and the process of PTG.

Furthermore, the main-effect hypothesis of social support (Cohen and Wills, 1985) suggests that social support plays an independent role in reducing pressure. Studies based on this model indicate social support is a protective factor for PTSD (Jia et al., 2015; Zhou et al., 2017). Calhoun and Tedeschi (2006) PTG model suggests social support can provide trauma survivors with a safe environment where they can talk freely with others about traumatic experiences and associated emotions. Therefore, social support can lead individuals to reframe traumatic experiences and reconstruct worldviews after trauma, thus potentiating PTG (Zhou et al., 2014). Additionally, social-cognitive process theory (Lepore et al., 2000) suggests a socially supportive environment encourages an active cognitive processing of stressful experiences, leading to resolution and integration of trauma-related material that results in positive psychological adjustments (including PTG). Such models account for positive links between social support and PTG (Zhou and Wu, 2016).

In general, gratitude and social support are both positive psychological feelings caused by survivor guilt and might evoke a secure attachment style that tends to encourage people to explore creative possibilities (Mikulincer and Shaver, 2007). Therefore, adolescents with high levels of gratitude and social support are likely to experience less distress and more growth after a traumatic event (Zhou et al., 2017, 2018).

In 2013, the Ya’an region of southwestern China was hit by a destructive earthquake measuring 7.0 on the Richter scale. The present study surveyed adolescent survivors of Lushan county three and a half years after the Ya’an earthquake to examine how survivor guilt predicts PTSD and PTG among adolescent survivors by including gratitude and social support as simultaneous predictors.

## Materials and Methods

### Participants and Procedures

In the present study, 706 adolescents were selected from one middle school and one high school in Lushan county, Sichuan province, China. The mean age of the participants was 14.12 years (*SD* = 1.65), and the range was 11.0–18.0 years, 380 (53.8%) were female and 325 (46.1%) were male, one participant did not report the gender. All the participants experienced the Ya’an earthquake three and a half years ago, about 20% of the participants were trapped or injured in the earthquake.

The Ethics of Committee by the Faculty of Psychology at Beijing Normal University and the principals of the participating schools approved this study. All the participants signed a written informed consent. In consideration of all participants in the current study were juveniles under the age of 18, written informed consent was obtained from the parents of all participants before the survey. All the participants were asked to complete the measures that assessed traumatic exposure, survivor guilt, gratitude, social support, PTSD and PTG. The assessment was performed by trained individuals with a Master’s degree in psychology.

### Measures

#### Trauma Exposures Questionnaire

We used Trauma Exposures Questionnaire (Wu et al., 2013) to measure the traumatic experiences in the earthquake of adolescent survivors (e.g., “The earthquake injured relatives and friends”). It is an 18-items scale, each of the items is rated on a 3-point scale. In this study, the internal reliability of the questionnaire was good (α = 0.82).

#### The Interpersonal Guilt Questionnaire

The Interpersonal Guilt Questionnaire (O’Connor et al., 1997) is a 67-items scale, which designed to measure five subcategories of guilt: survivor guilt (22 items), omnipotent guilt (14 items), separation guilt (15 items) and self-hate (16 items). In the current study, we only used the subcategories of survivor guilt (e.g., “It makes me very uncomfortable to receive better treatment than the people I am with”) Each item is scored on a 5-point scale ranging from 1 (*completely disagree*) to 5 (*completely agree*). In this study, the subscale demonstrated well an internal consistency (α = 0.82).

#### Gratitude Questionnaire

Gratitude was measured by Chinese version of the Gratitude Questionnaire (Wei et al., 2011). The original Gratitude Questionnaire developed by McCullough et al. (2002) and included six items (e.g., “I have so much to be grateful in my life”). Participants were asked to rate each item by the Likert Scale ranging from 0 (*completely disagree*) to 6 (*completely agree*). In the present study, the revised GQ-6 had good internal consistency (α = 0.83).

#### Social Support Questionnaire

This questionnaire developed by Zou (1999) and included 20 items (e.g., “Giving me some suggestion on solving problems”). There are five subcategories in this scale as follows: emotive support, instrumental support, companion, affirmative evaluation, and intimacy. All the items are rated on a 5-point Likert scale that ranges from 0 (completely disagree) to 4 (completely agree). In this study, the internal reliability of the questionnaire was good (α = 0.96).

### PTSD Checklist for DSM-5

PTSD was measured by PTSD Checklist for DSM-5 (Weathers, 2013). This measure is a 20-item self-report scale, designed to assess the occurrence and frequency of PTSD symptoms in relation to the most distressing event experienced by an individual (e.g., “The memories of the earthquake will intrude into my mind”). There are four subcategories in this scale as follows: intrusions, negative cognition and emotion alteration, avoidance, and hyper-arousal. The responses were made on a 4-point scale ranging from 0 (*not at all*) to 3 (*almost every week*). In this sample, the internal reliability of the scale was good (α = 0.91).

### Posttraumatic Growth Inventory

The Chinese version of Posttraumatic Growth Inventory (Zhou et al., 2014) was used to measure PTG (e.g., “I have found a new way for my life”), which was the revised version based on the Posttraumatic Growth Inventory (Tedeschi and Calhoun, 1996). The revised PTGI includes the following three subscales for a total of 22 items: perceived changes in self, changed the sense of relationships with others and changed the philosophy of life. The responses were provided on a 6-point scale, ranging from 0 (*no change*) to 5 (*to very great degree of change*). This questionnaire has good reliability for samples of Chinese adolescents after an earthquake (Zhou et al., 2014). In this sample, the internal reliability of the scale was good (α = 0.93).

### Data Analysis Strategies

All analyses were conducted by SPSS 22.0 and Amos 17.0. Full-information maximum likelihood (FIML) estimates were employed to impute missing data for this variable. The fit of the models was evaluated using the following four indices (Wen et al., 2004): chi-square test of model fit (χ^2^/df ≤ 5, acceptable); the comparative fit index (CFI ≥ 0.90, acceptable), the Tucker-Lewis index (TLI ≥ 0.90, acceptable), and the root mean square error of approximation (RMSEA ≤ 0.08, acceptable).

Considering the importance of traumatic exposure in the process of development of PTSD and PTG, thereby, the traumatic exposure were controlled in the model for examining the effect of survivor guilt, gratitude, social support on PTSD and PTG.

## Results

### Descriptive Statistics and Correlations

Table 1 shows that traumatic exposure were significantly associated with PTSD; survivor guilt was related significantly to gratitude, social support, PTSD and PTG; gratitude was related significantly to social support, PTSD and PTG; social support was related significantly to PTG but not PTSD; whereas the correlation between PTSD and PTG was no significant.

**Table 1:** Descriptive statistics, and correlations for key variables (*N* = 706).

Variables	*M*	*SD*	1	2	3	4	5	6
1. Traumatic exposure	20.94	4.43	1					
2. Survivor guilt	66.45	8.34	0.05	1				
3. Gratitude	25.11	6.55	−0.03	0.19^∗∗∗^	1			
4. Social support	46.34	16.99	0.06	0.18^∗∗∗^	0.47^∗∗∗^	1		
5. PTSD	13.87	10.01	0.19^∗∗∗^	0.34^∗∗∗^	−0.10^∗∗^	−0.02	1	
6. PTG	53.23	22.59	0.01	0.18^∗∗∗^	0.30^∗∗∗^	0.36^∗∗∗^	0.02	1

*PTSD, posttraumatic stress disorders; PTG, posttraumatic growth. ^∗∗∗^p < 0.001; ^∗∗^p < 0.01.*

### Structural Equation Model Analyses

We first estimated the model fit of the measurement model that included three latent variables of social support, PTSD and PTG. The social support latent variable has five subscales, the PTSD latent variable has four subscales, and the PTG latent variable has three subscales. The measurement model revealed a satisfactory fit to the data: χ^2^/df = 4.618, CFI = 0.962, TLI = 0.947, RMSEA (90% CI) = 0.072 (0.061 to 0.083).

We then built a direct effects model, which demonstrates that survivor guilt has direct effects on PTSD and PTG. The direct effects model demonstrated a good fit: χ^2^/df = 4.821, CFI = 0.953, TLI = 0.934, RMSEA (90% CI) = 0.073 (0.063 to 0.084). The direct effects model shows that survivor guilt was a significant positive predictor of PTSD (β = 0.354, 95% CI = 0.212 to 0.496) and PTG (β = 0.223, 95% CI = 0.096 to 0.351).

**FIGURE 1:**
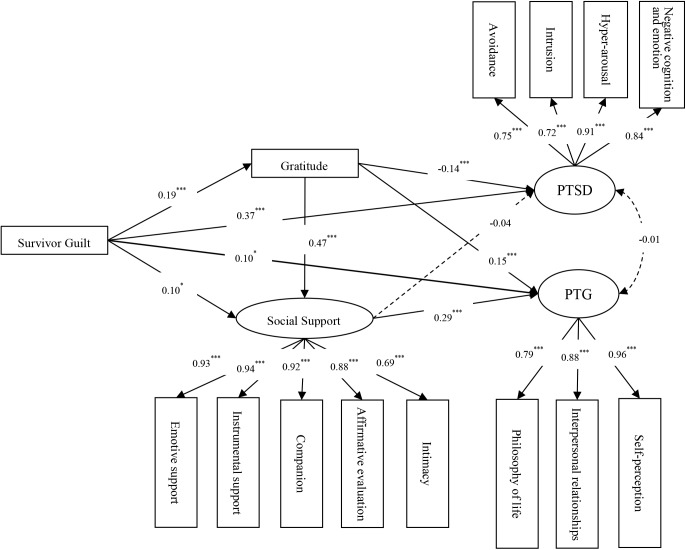
The multiple indirect effects model. PTSD, posttraumatic stress disorders; PTG, posttraumatic growth. ^∗∗∗^*p* < 0.001; ^∗^*p* < 0.05.

After controlling for traumatic exposure and based on the direct effect model, we inserted both gratitude and social support as mediating variables between survivor guilt and PTSD/PTG. Moreover, based on the broaden-and-build theory of positive emotions (Fredrickson, 2001), we added a path from gratitude to social support and established a multiple indirect effects model. The multiple indirect effects model depicted in Figure 1 demonstrated a good fit: χ^2^/df = 3.916, CFI = 0.961, TLI = 0.951, RMSEA (90% CI) = 0.064(0.058 to 0.071).

Next, we were using the bias-corrected bootstrap method to evaluate the significance levels of the multiple indirect effects model. The results showed that survivor guilt had an effect on PTSD and PTG through gratitude, which means that gratitude partly mediated the relation of survivor guilt with PTSD and PTG (β = −0.027, 95% CI = −0.049 to −0.005; β = 0.029, 95% CI = 0.008 to 0.051). Survivor guilt affected PTG but not PTSD through social support, which means that social support partly mediated the relation between survivor guilt and PTG (β = 0.029, 95% CI = 0.006 to 0.053) but not the relation between survivor guilt and PTSD (β = −0.004, 95% CI = −0.028 to 0.021). Additionally, survivor guilt affected PTG (β = 0.026, 95% CI = 0.005 to 0.054) but not PTSD (β = −0.003, 95% CI = −0.027 to 0.019) by social support following gratitude. Furthermore, the relation between PTSD and PTG was no significant.

## Discussion

To the best of our knowledge, this is the first study to examine the roles of survivor guilt, gratitude and social support simultaneously in predicting PTSD and PTG. After controlling for traumatic exposure, this study found that survivor guilt had a direct positive effect on PTSD and PTG, which was consistent with previous studies (Owens et al., 2008; Dekel et al., 2016). This result indicated that survivor guilt has a double-edged sword effect. On the one hand, survivor guilt is a negative feeling that may impede the emotional processing of fear and cause survivors to ruminate over traumatic experiences, which maintains the negative psychological reactions such as PTSD after trauma (Ehlers and Steil, 1995; Pugh et al., 2015). On the other hand, survivor guilt is regarded as a moral emotion and can play an adaptive role that may foster PTG in self-perception, interpersonal relationships and life philosophy, such as feeling more confident and finding a deeper understanding of the meaning of life (Tangney and Tracy, 2012).

In addition, this study found that survivor guilt had a negative effect on PTSD through gratitude. This result indicated that although guilt is a negative feeling for individuals, guilt as a moral emotion can cause the individual to promote gratitude, which can then reduce symptoms of PTSD. Furthermore, survivor guilt positively predicted PTG via gratitude. Consistent with the broaden-and-build theory of positive emotions (Fredrickson, 2001), gratitude can broaden thought-action repertoires, help survivors bounce back from negative emotional experiences, and eliminate the physiological effects of negative emotions. Shortly after the earthquake, adolescents were more likely to focus on the negative aspects of traumatic experiences (Scher et al., 2005), but adolescents who were grateful to others will have more opportunities to realize positive changes over time (Zhou and Wu, 2016).

This study also found that survivor guilt can predict PTG through the mediating role of social support. Guilt can encourage individual engagement in compensatory behavior and generate preoccupation with the well-being of others (Baumeister et al., 1994; Carnì et al., 2013). As a result, individuals with higher levels of survivor guilt may have better interpersonal relationships. The result of the relation between social support and PTG is consistent with previous studies (Zhou and Wu, 2016; Jia et al., 2017).

Social support encourages individuals to be more willing to share their emotions with others, encourages establishing good relationships, and promotes PTG (García et al., 2015). However, this study found that survivor guilt could not predict PTSD via social support. One possible reason is that earthquake survivors who are always protected by others’ social support cannot develop effective coping skills; thus, it is difficult for survivors to relieve negative posttraumatic reactions, such as PTSD (Zhou and Wu, 2016).

Another interesting finding of the present study states that survivor guilt can affect PTG via gratitude through social support. According to the moral emotion hypothesis of gratitude (McCullough et al., 2001), gratitude can improve an individual’s moral motivation, and moral motivation can prompt individuals to have more prosocial behavior toward others, which contributes to building a beneficial relationship and increases others’ support for themselves.

However, it is important to note that survivor guilt does not always promote gratitude and social support for some individuals. Baumeister et al. (1994) suggested survivor guilt can arise from personal transgressions or empathizing with others’ misfortune. For empathy-based survivor guilt, individuals are more easily prone to the reasoning that one’s advantage was at the expense of someone else (Blacher, 2000). Thus, empathy-based survivor guilt might promote more gratitude and social support. The different forms of survivor guilt should be distinguished in clinical practice. Clinicians should pay more attention to the survivor guilt arise from personal transgressions, which may lead to more negative psychological responses.

It is worth mentioning that this study did not find a significant association between PTSD and PTG in the structural equation modeling. PTSD and PTG can co-exist in traumatized people, which is a possibility that can shed light on which factors might predict either or both outcomes (Kleim and Ehlers, 2009; Dekel et al., 2011). For instance, some factors that can elicit PTSD have also been suggested as facilitators of PTG (i.e., traumatic exposure; Linley and Joseph, 2004). Differently, some factors can positively predict one of the two outcomes but negatively predict the other (i.e., a sense of meaning and purpose in life; Lowe et al., 2013). There is also another situation: the same factors are associated with each outcome in a reverse manner (i.e., perception of control; Dekel et al., 2011). Consequently, whether PTSD and PTG are positively related depends on which variables are controlled. The study found that PTSD and PTG are independent of each other in controlled for trauma exposure, guilt, gratitude, and social support. Moreover, a new study found both PTSD and PTG had a significant positive correlation in the general affected area after the Wenchuan earthquake. However, in the worst-hit areas, the relation between PTSD and PTG had a significant negative correlation, suggesting the extent of trauma exposure may affect the relation between PTSD and PTG (Du et al., 2018). Posttraumatic stress symptoms in adolescents were low three and a half years after the earthquake. Future studies could explore the relation between PTSD and PTG at different time points after the earthquake.

## Conclusion

In conclusion, these findings support the assumption that the influence mechanisms of PTSD and PTG are different (Chan et al., 2011; Zhou et al., 2017). Some predictive factors such as survivor guilt have a direct and positive effect on both PTSD and PTG, but gratitude and social support play different roles in the effect of guilt on PTSD and PTG. The results suggest PTSD and PTG are independent posttraumatic reactions (Linley and Joseph, 2004).

Several limitations exist within the current study that should be acknowledged. First, the present study was a cross-sectional study, which limits the utility of causal inferences, and future research should focus on this issue using a longitudinal design. Second, self-report measures are also a limitation of this study. In future studies, researchers should consider gathering data through multiple methods.

Notwithstanding these limitations, this study explores the relation between survivor guilt, gratitude, social support, PTSD, and PTG. The study indicated that survivor guilt has a double-edged sword effect which can improve levels of both PTSD and PTG, and gratitude and social support play an important mediating role in the effect of survivor guilt on PTSD and PTG.

This study also highlights important implications for adolescent survivors of the Ya’an earthquake from an intervention and health-enhancement perspective. The assessment for the adaptation of adolescents with traumatic experiences should incorporate both positive and negative indices of adjustment. Clinical efforts should focus on the exertive positive effect of survivor guilt and improving gratitude and social support. For example, when a survivor feels guilty about what he or she did during the earthquake, school psychologists or parents should guide the individual to appreciate those who helped them and encourage them to build better relationships with others. In this way, adolescents can positively reframe the traumatic event and mitigate negative posttraumatic effects to contribute to positive posttraumatic growth.

## Author Contributions

WW developed the study design, participated in and supervised data collection, performed the statistical analysis, and drafted the manuscript. XW conceived the study and revised the manuscript critically for important intellectual content. YT participated in and supervised data acquisition, modified the manuscript. All authors gave their final approval of the current version of the manuscript.

## Conflict of Interest Statement

The authors declare that the research was conducted in the absence of any commercial or financial relationships that could be construed as a potential conflict of interest.
